# Alveolar Bone and Epithelial Attachment Status following Two Different Closed-eruption Surgical Techniques for Impacted Maxillary Central Incisors

**DOI:** 10.5005/jp-journals-10005-1532

**Published:** 2018-08-01

**Authors:** Elia Sfeir, Mona Gholmieh, Zouhair Skaf, Ayman Mourad

**Affiliations:** 1 Professor, Department of Pediatric Dentistry, School of Dentistry, Lebanese University, Beirut, Lebanon; 2 Assistant Professor, Department of Pediatric Dentistry, School of Dentistry, Lebanese University, Beirut, Lebanon; 3 Chef de Clinique, Department of Orthodontics, School of Dentistry, Lebanese University, Beirut, Lebanon; 4 Associate Professor, Department of Mathematics, Sciences Faculty, Lebanese University, Beirut, Lebanon

**Keywords:** Closed-eruption surgical technique, Discontinued traction, Impacted, Periodontal status, Upper central incisor.

## Abstract

**Aim:**

Two eruption surgical techniques are commonly described for the treatment of upper impacted central incisors (ICIs): Open and closed. Currently, the closed-eruption surgical technique (CEST) is the most commonly used, as it allows for the best esthetic and periodontal results.

The aim of this study was to determine the effect of traction discontinuation on maxillary central incisor sulcal depth and alveolar bone ridge levels compared with contralateral incisors, when CEST is used.

**Materials and methods:**

Our study involved 28 unilateral impacted maxillary central incisors treated by CEST. Thirteen teeth were subjected to traction interruption for a month at the time of emergence of the crown, while 15 teeth underwent continuous traction. One year after treatment, periapical digital X-rays, anterosuperior cone beam computerized tomography (CBCT) scanning, and periodontal probing of the ICIs and contralateral central incisors (CCIs) were performed. Student’s t-test was used to study whether a statistically significant difference between continuous and interrupted tractions takes place while using the CCI measurements as reference.

**Results:**

There was a statistically significant difference between the two techniques only for the following measurements: Mesial probing (p-value 0.039352), labial bone level (p-value 2.58E-08), and palatal bone level (p-value 2.56E-06).

**Limitations:**

A larger sample size and longer term follow-up are needed to draw more robust conclusions.

**Conclusion:**

A temporary discontinuation during traction of the tooth appears to positively impact treatment outcome on ICIs.

**Clinical significance:**

• The CEST leads to the best periodontal status for ICIs.

• The discontinuation of traction at the emergence of the tooth allows the supracrestal fibers to insert into the cement in a proper way.

**How to cite this article:** Sfeir E, Gholmieh M, Skaf Z, Mourad A. Alveolar Bone and Epithelial Attachment Status following Two Different Closed-eruption Surgical Techniques for Impacted Maxillary Central Incisors. Int J Clin Pediatr Dent 2018;11(4):317-322.

## INTRODUCTION

Impaction of maxillary central incisors is part of the eruption failure of permanent teeth and remains relatively rare with a frequency ranging from 0.06 to 0.2%.^1^ Numerous causes can be responsible for impaction, including the presence of odontomas, supernumerary teeth, dentiger-ous cysts, history of trauma of the temporary incisor, or root dilaceration in the incisor.^2-7^ After addressing the cause, 63.6% of impacted teeth may proceed to their normal eruption.^8,9^ However, many impacted ones still do not erupt.^10^ Thus, a second surgical procedure is required, followed by orthodontic traction, in order to bring the tooth into the arch.

Two surgical techniques are described in the orthodontic literature for the resolution of impacted maxillary central incisors. First, the CEST, which involves fully replacing the mucoperiostal flap in its former position after an attachment has been bonded to the impacted tooth.^10-12^ With the CEST, superior outcomes are obtained in terms of the gingival, periodontal, and pulp status. Second, the open-eruption surgical technique (OEST) involves suturing a full thickness of the flap apically, while leaving a portion of the labial surface of the incisor uncovered.^13^

For both surgical techniques, it is widely agreed there should be sufficient space in the arch before undertaking any traction.^14-16^ In addition, researchers have studied the consequences of these surgical treatments on tooth periodontium and shade. Some authors found that there was no difference in the periodontal status or shade when compared with the contralateral tooth with the use of OEST.^17,18^ In contrast, others showed the superiority of CEST on the quality of the periodontium and the level of the alveolar ridge, as well as the length and shade of the crown.^12,14,19,20^ Furthermore, researchers reported a positive impact with the use of the CEST on the periodon-tium, in terms of the depth of the sulcus, the level of the alveolar ridge, and the gingival contour.^12^

In the case of the CEST, authors proposed to temporarily discontinue traction when the tooth is at the stage of emergence, so the supracrestal fibers can insert into the cementum to mimic the physiological conditions of erup-tion.^21^ Thus, interrupting traction is considered when the cementoenamel junction, identified using a periodontal probe, has crossed the mucogingival line.^21^

The aim of our study was to determine the effects of discontinuation of traction during CEST use on the sulcus depth and alveolar bone ridge levels of maxillary central incisors when compared with contralateral incisors.

## MATERIALS AND METHODS

Patients were recruited from the Department of Pediatric Dentistry at the University and from the private practice of former university dental residents. The study has been conducted in accordance with the Declaration of Helsinki. Inclusion criteria were limited to cases where a single maxillary central incisor was impacted. Impacted maxillary central incisors with root dilacerations or sharp angulation were excluded from the study. The study sample consisted of 28 unilateral impacted maxillary central incisors due to an obstacle, insufficient space or insufficient eruption potential.

Each of the cases was examined, evaluated, and operated on by two of the authors (ES and MG) in the same session. Comparisons were made between the previous ICI and the normally erupted CCI. A total of 28 completely erupted contralateral maxillary central incisors represented the control group. The treatment group comprised 7 girls and 21 boys, and their age ranged between 8 and 10 years. Conservative treatments and oral hygiene motivation were undertaken before the beginning of the interceptive orthodontic treatment. The 28 ICIs were randomly assigned to discontinuous or continuous traction for a final count of 13 to 15 respectively, regardless of the age and sex of the child.

A preliminary explanation of the purpose of the study and an assurance of wearing a lead protective apron when taking X-rays were given prior to the written consent of the parents.

All surgical interventions were performed by single operator (ES) using the CEST, as described previously, with one incision along the gingival crest and another vertical incision made mesially to the impacted tooth along the labial frenum.^21,22^ Where required, sufficient space for eruption of the impacted tooth was created. A light traction of 1 ounce (30 gm), measured with a force gauge (Leone Spa, Firenze, Italy), was applied through an elastic chain from the bonded attachment at the buccal surface of the tooth to the orthodontic appliance. The orthodontic treatment was performed by a single operator (ZS).

A total of 13 impacted maxillary central incisors were subjected to traction interruption for a month after crown emergence while the remaining 15 impacted maxillary central incisors underwent continuous traction.

**Fig. 1:**
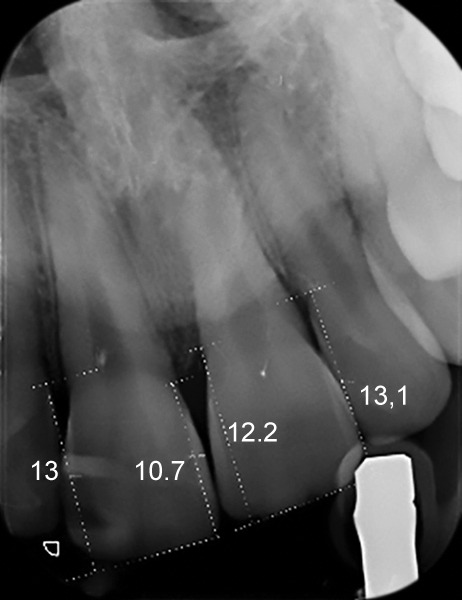
Measurements were digitally calculated from the incisor edge to the top of the alveolar ridge on each side

**Fig. 2:**
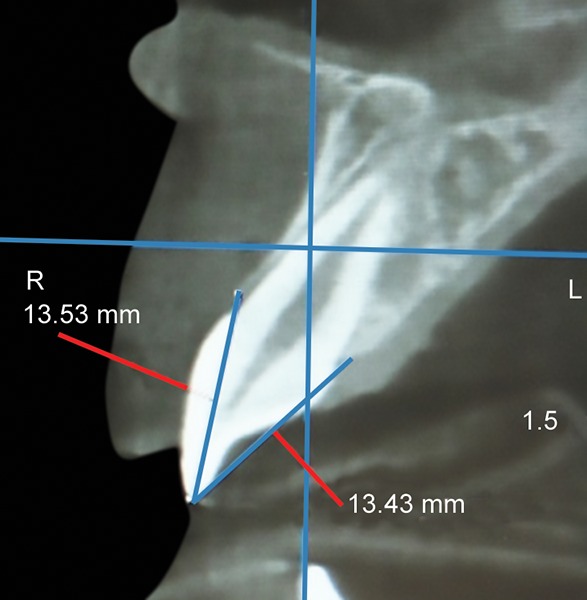
Measurements of the labial and palatal alveolar ridge from the incisor edge to the top of the alveolar ridge on each side

Twelve months after the tooth had reached its final position, the following examinations were performed by the same two authors (ES and MG) conjointly and after reaching a consensus: (1) Periapical digital X-rays using the VistaScan Mini Easy Plus (Di) rr Dental, Bietingheim-Bissigen Germany) for the evaluation of mesial and distal alveolar ridges of the ICIs and those of the CCIs. Measurements were taken after calibration of each X-ray. The EndoRay II (DENTSPLY International Inc., 1212 Abbott Drive, Elgin, Illinois, USA) was used with a fixed distance of 7 cm between the cone and the film in all cases. Measurements were digitally calculated from the incisal edge to the top of the alveolar ridge on each side (Fig. 1); (2) anterosuperior CBCT (PaX-i3D software, Vatech, Secaucus, New Jersey, USA) scans to evaluate the buccal and palatal alveolar ridge levels of ICIs and CCIs, with the incisal edge as reference (Fig. 2); and (3) periodontal probing (using a Goldman Fox periodontal probe) of the ICIs and CCIs on all four sides of each tooth. These measurements were rounded to the nearest 0.5 mm and fixed at the beginning of gum whitening (calibrated pressure between 10 and 20 gm between the two operators).^23^

### Statistical Analysis

In order to compare the discontinuous and continuous traction techniques, first, we calculated the difference between the impacted teeth and the control contralat-eral teeth, for each of the following eight measurements: Mesial probing, mesial bone level, labial probing, labial bone level, distal probing, distal bone level, palatal probing, and palatal bone level (Table 1). Second, we used the one-tailed Student’s t-test to compare the average differences of the two groups (discontinuous traction *vs* continuous traction). Two statistical tests have been applied, the first with the assumption that the two samples have the same variance and the second with the assumption that the two samples have different variances. A p-value <0.05 was considered statistically significant.

## RESULTS

The mean values ± standard deviation (SD) of both ICI and CCI measurements are presented in Tables 1 and 2 for the two techniques, i.e., continuous and discontinuous tractions. Similarly, the mean values ± SD of the difference between the ICI and CCI measurements are presented in Table 3.

Tables 4 and 5 summarize the results of the statistical analysis. We found a statistically significant difference between the discontinuous traction group and the continuous traction group only for the following measurements: Mesial probing (p-value 0.039352), labial bone level (p-value 2.58E-08), and palatal bone level (p-value 2.56E-06). Measurement results for the other five parameters were similar in both groups.

**Table 1:** **Table 1:** Mean value ± SD of difference between ICI and CCI

*Mean ± SD*		*Mesial probing*		*MBL*		*Labial probing*		*LBL*		*Distal probing*		*DBL*		*Palatal probing*		*PBL*	
M*		0.1923 ±		0.1846 ±		0.3462 ±		0.0769 ±		0.2308 ±		0.2769 ±		0.2692 ±		0.0846 ±	
		0.3125		0.4035		0.3028		0.1250		0.2492		0.5578		0.2492		0.1292	
M		0.4333 ±		0.2 ± 0.2394		0.4333 ±		0.4467 ±		0.4 ±		0.2267 ±		0.2333 ±		0.38 ±	
		0.3590				0.3091		0.1204		0.3266		0.2081		0.3590		0.1327	

MBL: Mesial bone level; LBL: Labial bone level; DBL: Distal bone level; PBL: Palatal bone level; M*: Discontinuous traction; M: Continuous traction

**Table 2:** **Table 2:** Mean values ± SD of ICI

*Mean ± SD*		*Mesial probing*		*MBL*		*Labial probing*		*LBL*		*Distal probing*		*DBL*		*Palatal probing*		*PBL*	
M*		2.6154 ±		12.2692 ±		2.4615 ±		13.2692±		2.6538 ±		12.4154 ±		2.7308 ±		13.1614 ±	
		0.2878		0.3851		0.2371		0.4321		0.2308		0.3958		0.2493		0.4270	
M		2.6333 ±		12.3133 ±		2.4667 ±		13.64 ± 0.1781		2.7 ± 0.2449		12.32 ±		2.6667 ±		13.5 ±	
		0.2867		0.2306		0.2867						0.2227		0.3496		0.1862	

MBL: Mesial bone level; LBL: Labial bone level; DBL: Distal bone level; PBL: Palatal bone level; M^*^: Discontinuous traction; M: Continuous traction

**Table 3:** **Table 3:** Mean values ± SD of CCI

*Mean ± SD*		*Mesial probing*		*MBL*		*Labial probing*		*LBL*		*Distal probing*		*DBL*		*Palatal probing*		*PBL*	
M*		2.4230 ±		12.0846 ±		2.1154 ±		13.1923 ±		2.4231 ±		12.1385 ±		2.4615 ±		13.0769 ±	
		0.3309		0.6871		0.2107		0.4984		0.2665		0.5400		0.2371		0.4264	
M		2.2 ±		12.1133 ±		2.0333 ±		13.1933 ±		2.3 ±		12.0933 ±		2.4333 ±		13.12 ±	
		0.24494		0.3896		0.1247		0.1692		0.2449		0.3316		0.1700		0.1558	

MBL: Mesial bone level; LBL: Labial bone level; DBL: Distal bone level; PBL: Palatal bone level; M^*^: Discontinuous traction; M: Continuous traction

**Table 4:** **Table 4:** One-tailed t-test for two independent means

		*Mesial probing*		*MBL*		*Labial probing*		*LBL*		*Distal probing*		*DBL*		*Palatal probing*		*PBL*	
p-value		0.040749		0.452709		0.237774		1.91E-08		0.077105		0.621307		0.613609		2.47E-06	
Significance		Yes		No		No		Yes		No		No		No		Yes	

The two samples have the same variance, significance level: 0.05; MBL: Mesial bone level; LBL: Labial bone level; DBL: Distal bone level; PBL: Palatal bone level

**Table 5:** **Table 5:** One-tailed t-test for two independent means

		*Mesial probing*		*MBL*		*Labial probing*		*LBL*		*Distal probing*		*DBL*		*Palatal probing*		*PBL*	
p-value		0.039352		0.454536		0.237588		2.58E-08		0.073425		0.613987		0.61641		2.56E-06	
Significance		Yes		No		No		Yes		No		No		No		Yes	

The two samples dont have the same variance, significance level: 0.05; MBL: Mesial bone level; LBL: Labial bone level; DBL: Distal bone level; PBL: Palatal bone level

**Figs 3A and B:**
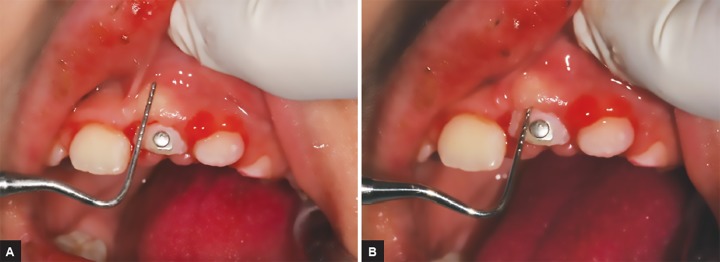
(A) Probing to determine the level of the supracrestal fibers. (B) Transposition of the measurement to determine if the cementoenamel junction has crossed the mucogingival line. At this time the interruption of the traction is considered

## DISCUSSION

To our knowledge, this is the first study that uses new imaging techniques (VistaScan Mini Easy Plus and CBCT) to investigate the effect of discontinued traction on the periodontal status of a retained maxillary central incisor, compared with the contralateral incisor, by evaluating the sulcus depth and the level of the alveolar ridge of the treated ICI and its CCI (a split-mouth design study) by determining the level of the buccal and palatal alveolar ridges of the examined teeth.^24^

In general, study findings reported on periodontal status in cases of maxillary ICIs and canines have been conflicting, probably because of the use of different surgical methods and the small number of cases studied.^12-14,17-19^

In this study, we adopted the CEST as our surgical approach, and, in all treated cases, we followed the same surgical protocol for the incisions. A horizontal incision along the gingival crest, together with a vertical incision mesially to the impacted tooth and distally to the labial frenum, prevents any traumatic effect to the periodontium of the adjacent teeth. Moreover, this procedure provides adequate access to the impacted tooth.

When the incisal edge of the ICI appears at the gin-gival crest, it marks the beginning of dental emergence. At this point, we can see that the probe penetrates along the junctional epithelium to the mucogingival line. This means that, at this stage of eruption, the gingival tissue is in contact with the enamel and cannot therefore be attached to the cementum. The collagen fibers can only be inserted on the root when the cementoenamel junction exceeds the mucogingival line.^22^ When the incisal edge has exceeded the level of the gingival crest, it is the emergence phase of the tooth itself. The supracrestal fibers are able to be inserted into the cement. At this stage, it is recommended to stop the traction of the tooth.^22^ It is based on the height of the gum and the crown that we can assess the reported level of the cementum surface with the mucogingival line (Fig. 3). During this period of migration, it is suitable to reproduce the physiological conditions of the eruption.

However, taking the root apices of the ICIs and CCIs as landmark points for the measurement of the alveolar ridge level can potentially influence the results as previously reported.^12^ A slight apical resorption or a deviation of the apex relative to the axis of the root can lead to an inaccurate comparison with the CCI. In our study, we designated the incisal edge as the landmark point for measurements, as the sizes of the crown of a right and left central incisor are usually superimposable. In addition, the software used with the new imaging machines is capable of generating data of very high accuracy.

In our study, results showing the mean difference in sulcus probing are in agreement with reports of some authors, although different from those of others (Table 3).^13,25^ In the case of discontinuing traction, our findings on the mean difference in sulcus probing are consistent with the results reported in another study on the CEST.^12^

Our results show that the mean difference in sulcus probing for the discontinuous traction group was smaller than, or almost equivalent to, the mean difference obtained for the continuous traction group. On comparing the depth of the mesial sulcus, in the discontinuation traction group, the value of this depth was significantly lower in the CCI than in the ICI (p-value <0.05), and this result is lower than those reported previously by others.^12,13^ It was not possible to compare these results with other studies where the discontinuing traction protocol was applied.^22^ However, this study shows that interrupting traction improves the level of epithelial attachment, especially for the mesial sulcus and, according to other authors, may reduce the possible higher risk of gingival recession.^13,25^ Moreover, the mean differences in the bone level mesially and distally between the ICI and CCI obtained here are in line with previous reports.^12,13,25^ Importantly, we found a statistically significant difference at the labial and palatal bone levels in the discontinuous traction group, with a net reduction in the bone height loss of the labial and palatal alveolar ridges when traction was discontinued temporarily.

Therefore, the proposal to interrupt traction at the emergence stage of the treated tooth could play a favorable role in the status of epithelial attachment.^22^ It is evident that, with this approach, there would be an improvement in the position of the labial and palatal alveolar crests of the treated tooth, hence, leading to a lower risk of recession or labial bone dehiscence. However, the overall clinical consequences of this conservative approach and orthodontic alignment of impacted incisors are minimal in the short term.

The results of this study cannot be easily compared with other studies using the same criteria. The individual effect of specific variables that could affect the ultimate result (such as etiology, treatment time, height of impac-tion) was not studied because of the small numbers involved in this study. An impacted incisor is considered to have lost its potential of eruption when it does not resume eruption after having eliminated the cause of retention. Caution is necessary when drawing definitive conclusions based on this study’s approach. Studies with larger sample sizes, longer term follow-up, and identification of impaction cause(s) are needed to better describe the benefits (or disadvantages) of traction discontinuation.

Still, it remains that discontinuing traction at the time of emergence of the tooth could be a beneficial factor for the periodontal status of the ICIs.

## CONCLUSION

The most commonly used technique for the treatment of ICIs involves a closed-eruption orthodontic surgical technique. Technical modifications and the use of a temporary interruption during tooth traction can result in better treatment outcomes. Further studies with longer follow-up are needed to draw firmer conclusions.

### Clinical Significance

- The CEST leads to the best periodontal status for ICIs.
- The discontinuation of traction at the emergence of the tooth allows the supracrestal fibers to insert into the cement in a proper way.

### Why This Article is Important to Pediatric Dentists

- This article highlights that interrupting traction improves the level of epithelial attachment, especially for the mesial sulcus.
- This study shows a net reduction in the bone height loss of the labial and palatal alveolar ridges when traction was discontinued temporarily.

### What This Article Adds

- Considering the importance of epithelial attachment and the alveolar bone level around the teeth, it is important to spread awareness among the dentists regarding the choice of the orthosurgical technique.
- The results described in this article can help dentists on the best technique for improved outcomes.
